# Detailed Analyses of Molecular Interactions between Favipiravir and RNA Viruses In Silico

**DOI:** 10.3390/v14020338

**Published:** 2022-02-07

**Authors:** Tatsuya Shirai, Mitsuru Sada, Takeshi Saraya, Daisuke Kurai, Soyoka Sunagawa, Haruyuki Ishii, Hirokazu Kimura

**Affiliations:** 1 Department of Respiratory Medicine, Kyorin University School of Medicine, Mitaka-shi 181-8611, Japan; dw57td@bma.biglobe.ne.jp (T.S.); saraya@ks.kyorin-u.ac.jp (T.S.); kuraida@aol.com (D.K.); h141@ks.kyorin-u.ac.jp (H.I.); 2 Department of Health Science, Gunma Paz University Graduate School, Takasaki-shi 370-0006, Japan; sada@paz.ac.jp (M.S.); s.sunagawa220@gmail.com (S.S.)

**Keywords:** favipiravir, human metapneumovirus, respiratory syncytial virus, mumps virus, measles virus, influenza virus, docking simulation

## Abstract

There are currently no antiviral agents for human metapneumovirus (HMPV), respiratory syncytial virus (RSV), mumps virus (MuV), or measles virus (MeV). Favipiravir has been developed as an anti-influenza agent, and this agent may be effective against these viruses in vitro. However, the molecular mechanisms through which the agent affects virus replication remain to be fully elucidated. Thus, to clarify the detailed molecular interactions between favipiravir and the RNA-dependent RNA polymerase (RdRp) of HMPV, RSV, MuV, MeV, and influenza virus, we performed in silico studies using authentic bioinformatics technologies. As a result, we found that the active form of favipiravir (favipiravir ribofuranosyl-5′-triphosphate [F-RTP]) can bind to the RdRp active sites of HMPV, RSV, MuV, and MeV. The aspartic acid residue of RdRp active sites was involved in the interaction. Moreover, F-RTP was incorporated into the growing viral RNA chain in the presence of nucleotide triphosphate and magnesium ions. The results suggested that favipiravir shows two distinct mechanisms in various viruses: RdRp active site inhibition and/or genome replication inhibition.

## 1. Introduction

Most antiviral agents inhibit genome replication or proteases and prevent viral entry of a target virus [1,2]. Nucleic acid analogs are an example of agents that inhibit viral genome replication [3]. These agents lead to the termination of genome replication or inhibit polymerase activity [3]. Human metapneumovirus (HMPV), respiratory syncytial virus (RSV), mumps virus (MuV), and measles virus (MeV) are associated with major common diseases [4,5,6]. It is therefore important to develop antiviral agents for these infectious diseases; however, there are no effective antiviral agents for them at present [4,6,7,8].

Favipiravir (6-fluoro-3-hydroxypyrazine-2-carboxamide, Avigan^®^) was synthesized as an anti-influenza agent and is classified as a nucleic acid analog [9]. Favipiravir has been reported to inhibit influenza virus genome replication [10,11]. Previous reports also showed that favipiravir inhibited other viral replications, including norovirus, Ebola virus, HMPV, RSV, MuV, and MeV, in cell culture systems [12,13,14,15]. However, detailed molecular interactions between the agent and viral proteins are not known. In silico approaches in drug discovery allow us to screen large numbers of compounds virtually in a short period of time, thus, reducing the initial cost of hit identification and increasing the likelihood of finding a drug candidate of interest.

Originally, these in silico approaches started with docking simulations to clarify the interaction between a compound and its molecular target [16]. At present, it is possible to go beyond mere docking and predict in silico the off-target effects and absorption, distribution, metabolism, and excretion properties of compounds [17,18]. However, docking simulations can now visually reveal more detailed molecular interactions for a broader range of molecular targets, owing to improvements in docking algorithms, homology modeling methods, and pharmacophore analysis [18,19,20].

These technologies may provide information regarding the molecular pharmacological effects between antiviral agents and variously functional viral proteins [16,21]. In addition, the identification of chemical features involved in the efficacy of drugs may not be only valuable in the search for new drugs but also provide a rational guide for the development of more promising new drugs [22]. With this background, to clarify the molecular pharmacology of favipiravir, we performed docking simulations between favipiravir and the RNA-dependent RNA polymerase (RdRp) of HMPV, RSV, MuV, MeV, and influenza virus.

## 2. Materials and Methods

### 2.1. Structure Retrieval and Sequence Analysis

We obtained three-dimensional (3D) structures of RdRp complex proteins (large protein and phosphoprotein) of HMPV (PDBID: 6U5O) [23] and RSV (PDBID: 6UEN) [24] from Protein Data Bank Japan (https://pdbj.org/, accessed on 17 May 2021) to prepare the docking simulation. To construct the homology model, the RdRp complex amino acid sequences of MuV and MeV (Protein ID: NP_054714.1, NP_054708.1, Protein ID: NP_056924.1, NP_056919.1, respectively) were downloaded from NCBI (https://www.ncbi.nlm.nih.gov/protein/, accessed on 21 May 2021). To compare molecular mechanisms with these viruses, we also obtained the RdRp amino acid sequences of influenza A/California/07/2009(H1N1) (Protein ID: YP_009118630.1, YP_009118628.1, YP_009118631.1) from NCBI. 

Subsequently, homologous sequences for the RdRp of MuV, MeV, and influenza H1N1 were retrieved using the BLAST (Basic Local Alignment Search Tool) software program (https://blast.ncbi.nlm.nih.gov/Blast.cgi, accessed on 21 May 2021) with the PDB [25]. Multiple sequence alignment was performed with CLUSTAL Omega web server (https://www.ebi.ac.uk/Tools/msa/clustalo/, accessed on 21 May 2021) to reveal the sequence conservation among the retrieved sequences [26]. Suitable template structures were determined by sequence identity to the target protein (MuV, MeV, and influenza H1N1).

The 3D structure of favipiravir ribofuranosyl-5′-triphosphate (F-RTP) (PubChem CID: 5271809), the active form of favipiravir, was retrieved from the PubChem database (https://pubchem.ncbi.nlm.nih.gov/, accessed on 28 May 2021).

### 2.2. Structural Modeling

The 3D structures of the RdRp proteins of MuV, MeV, and influenza H1N1 were not available. Therefore, we constructed homology models of them with the template structures using the MODELLER 9.23 software program (Windows version) [27]. The structural reliability of the generated models was assessed by a Ramachandran plot analysis using CooT 0.8.9.2 [28]. Then, energy minimization was performed for the most reliable structure using GROMOS96, which is implemented in Swiss PDB Viewer 4.1.0 [29].

We also generated a model of RdRp with nucleotide triphosphate (NTP) and magnesium ions to analyze molecular interactions among various RdRp, NTP, magnesium ions, and F-RTP. We conducted analysis of the binding of two magnesium ions to various RdRp proteins using a metal ion-binding site prediction and docking server (MIB) [30]. Subsequently, a protein-RNA docking analysis was performed using the HDOCK web-server [31]. We used the 3′-UUGUCUCUAG gene sequence, which plays a crucial role in the replication of vesicular stomatitis virus (VSV), as RNA for docking because VSV has served as a prototype to clarify the detailed mechanisms of transcription and replication of nonsegmented negative-strand RNA viruses [32].

The 3D structure of the gene sequence was constructed using AutoDockTools 1.5.6. The optimal model was determined by evaluating the docking score and the distance to the active site of each protein in both analyses. On the basis of previous reports, the following were identified as active sites essential for replication in the influenza H1N1 RdRp protein: Ser1160, Asp1161, and Asp1162 [33,34]. The RdRp active site of HMPV was formed by residues Gly744, Asp745, Asn746, and Gln747 [23]. The residues were conserved among the RdRp active sites of RSV, MuV, and MeV [35,36,37,38].

### 2.3. Protein-Drug Docking

Molecular docking studies were carried out using AutoDock Vina 1.1.2 according to the protocol [39]. Before docking studies, the proteins were prepared by adding polar hydrogen atoms, Gasteiger charges, and the generation of PDBQT files using AutoDockTools 1.5.6. The grid box for the analysis covered the whole protein. The default parameters of AutoDock Vina were used. The detailed processes of the docking studies were based on a previously reported procedure [39,40].

### 2.4. Post-Docking Analysis

After the docking simulation, we utilized PyMOL 2.3.4 to visualize the interaction between the ligand and the protein models in 3D. To ensure structural similarity, we excluded ligands associated with a root mean square deviation of >2 in comparison to values obtained before the docking analysis. The most favorable model was selected from the top 20 docking models based on the lower binding energy and the orientation of the ligand. Furthermore, interacting residues in the docking complex were detected, and a 2D diagram was generated using BIOVIA Discovery Studio Visualiser.

To validate the reliability of the present binding affinity prediction, we rescored and ranked these docking poses using PoseScore of the LigScore web service (https://modbase.compbio.ucsf.edu/poseandrank/, accessed on 12 January 2022). The PDBQT files of the docking poses were converted to mol2 files using Open Babel 2.4.0 [41].

## 3. Results

### 3.1. Multiple Sequence Alignment

**Figure 1:**
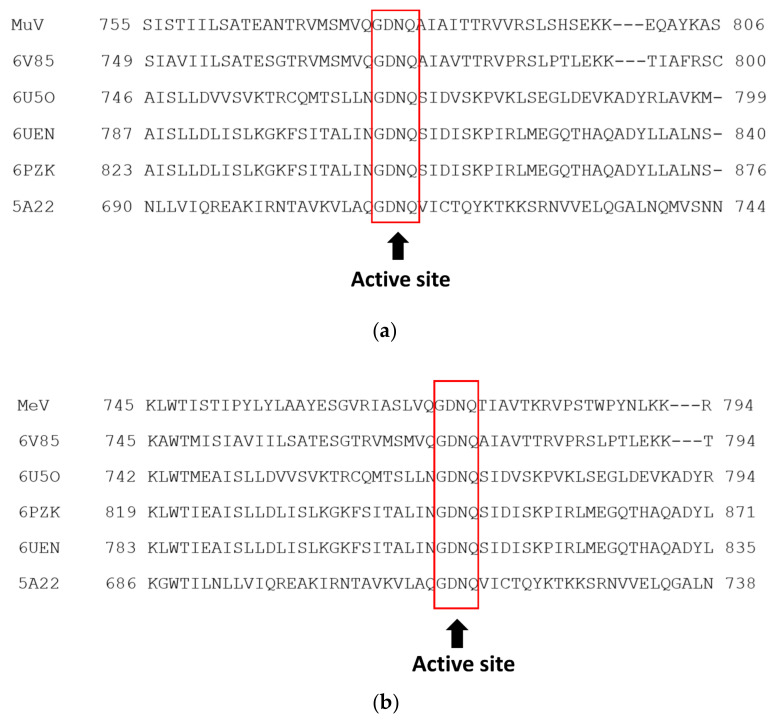

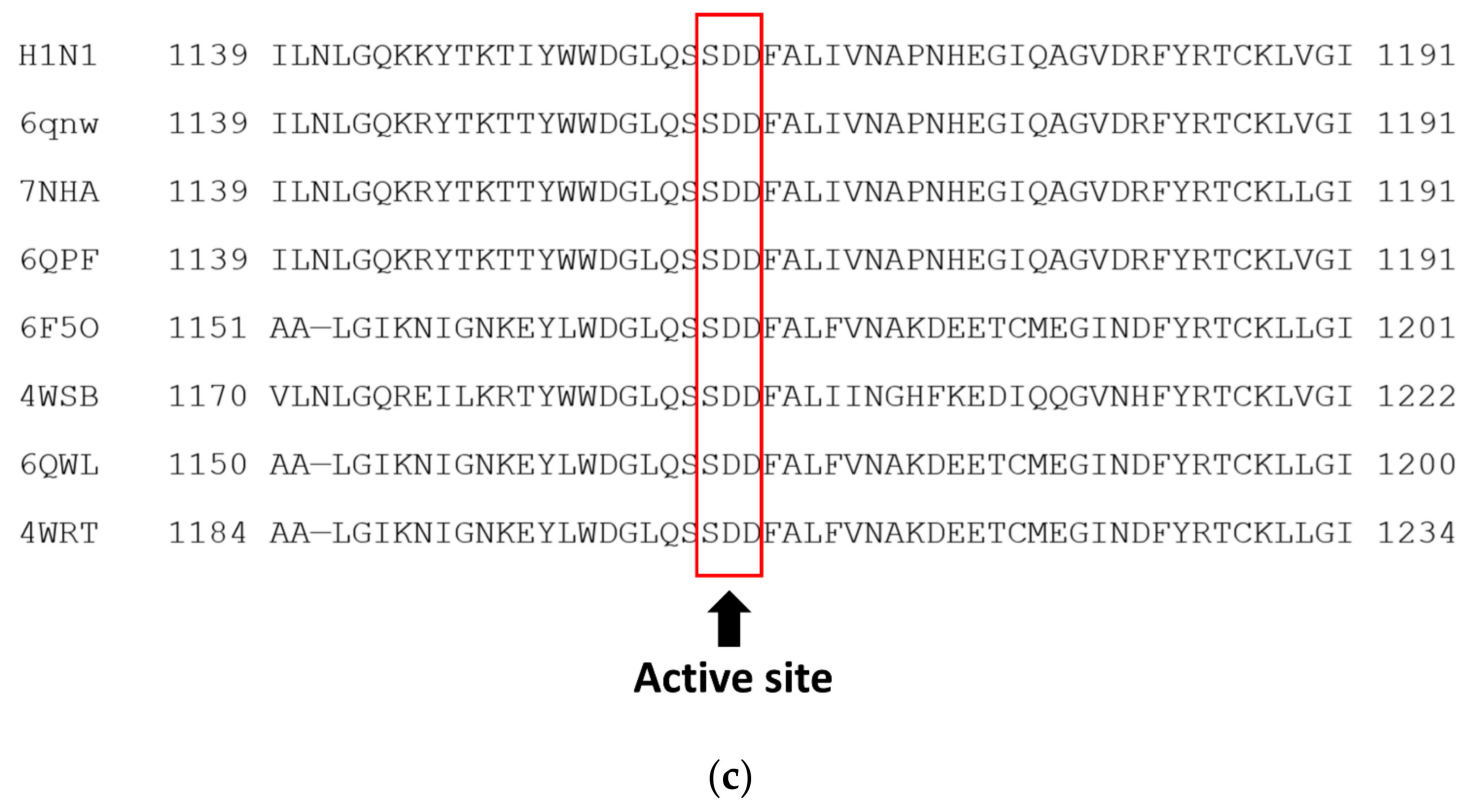
Diagram illustrating the multiple sequence alignment of RNA-dependent RNA polymerase (RdRp) amino acid sequences of (**a**) mumps virus (MuV), (**b**) measles virus (MeV), and (**c**) influenza H1N1 and other templates selected. The boxes indicate the active sites in RdRp.

The multiple sequence alignment of the RdRp amino acid sequences of MuV, MeV, and influenza H1N1 is shown in Figure 1. The active site residues were conserved between the target amino acid sequences and homologous sequences in each virus. We selected parainfluenza virus 5 RdRp complex protein (PDBID: 6V85) [42] for MuV and MeV and Influenza A/Northern Territory/60/1968(H3N2) (PDBID: 6QNW) [43] for influenza H1N1 as the suitable template structure. The percent sequence identity values against MuV, MeV, and influenza H1N1 were 55.4%, 29.9%, and 95.3%, respectively.

### 3.2. Molecular Interactions between F-RTP and Various RdRp Proteins

**Figure 2:**
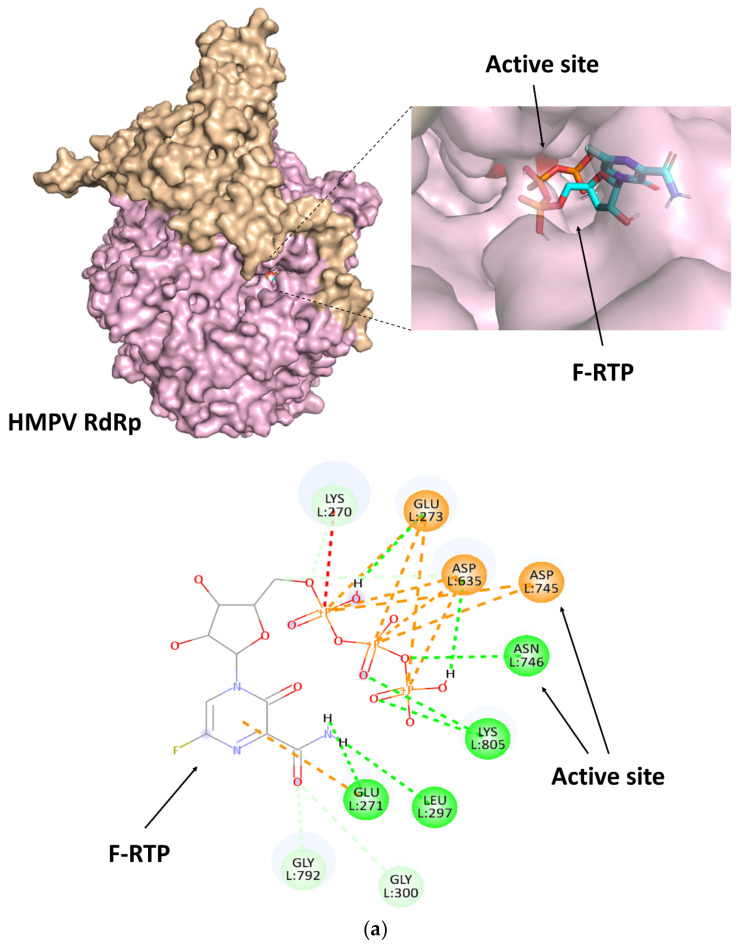

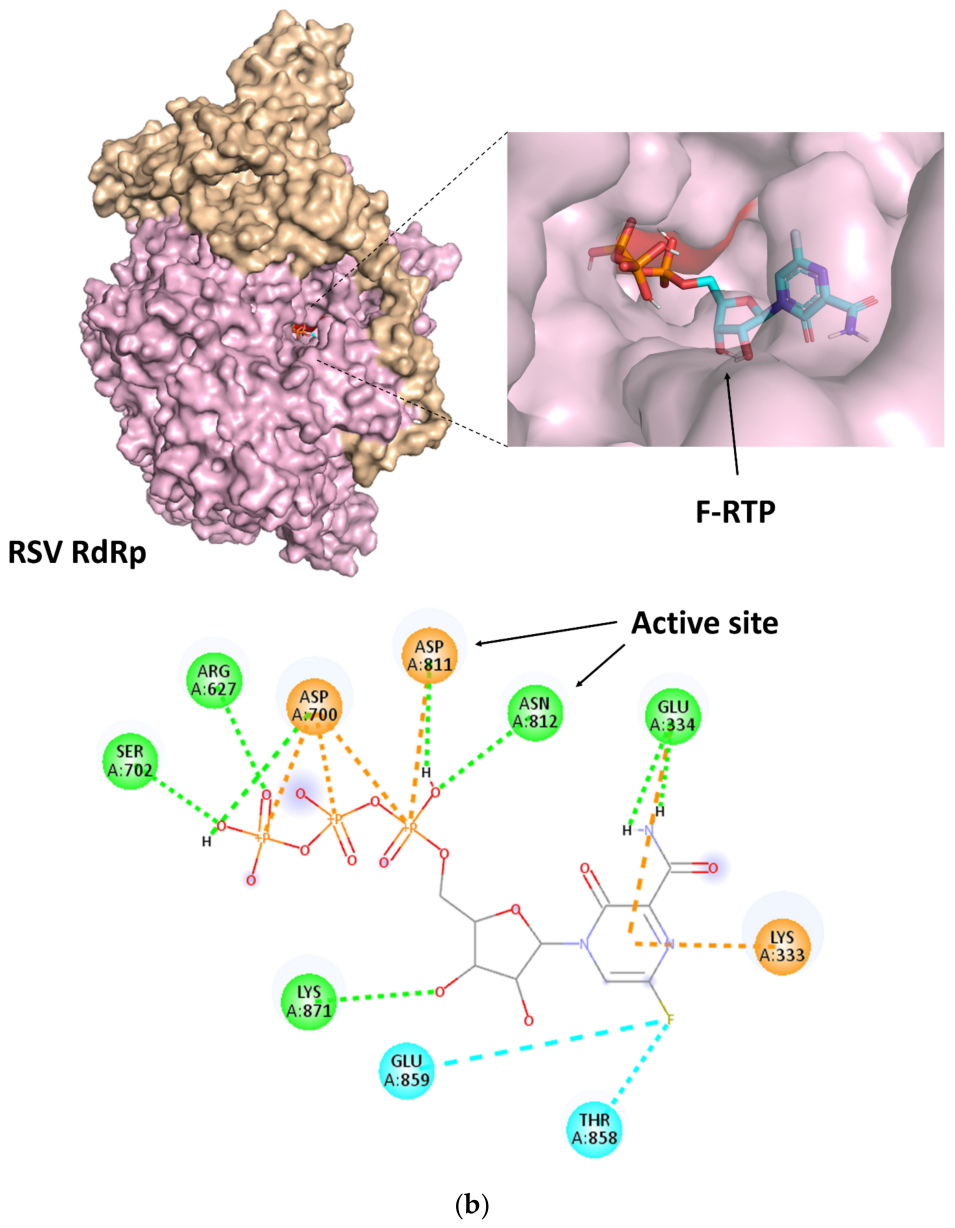

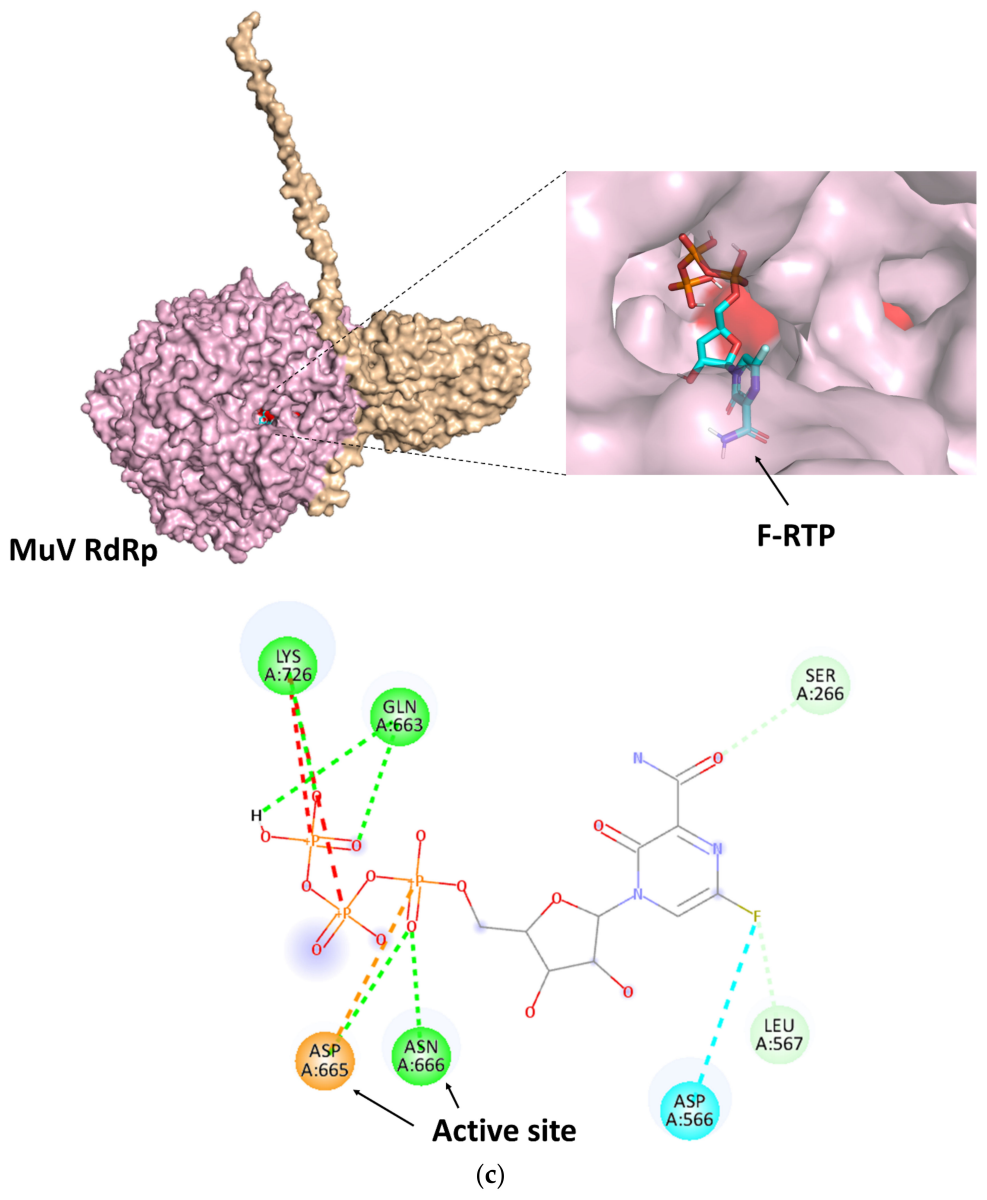

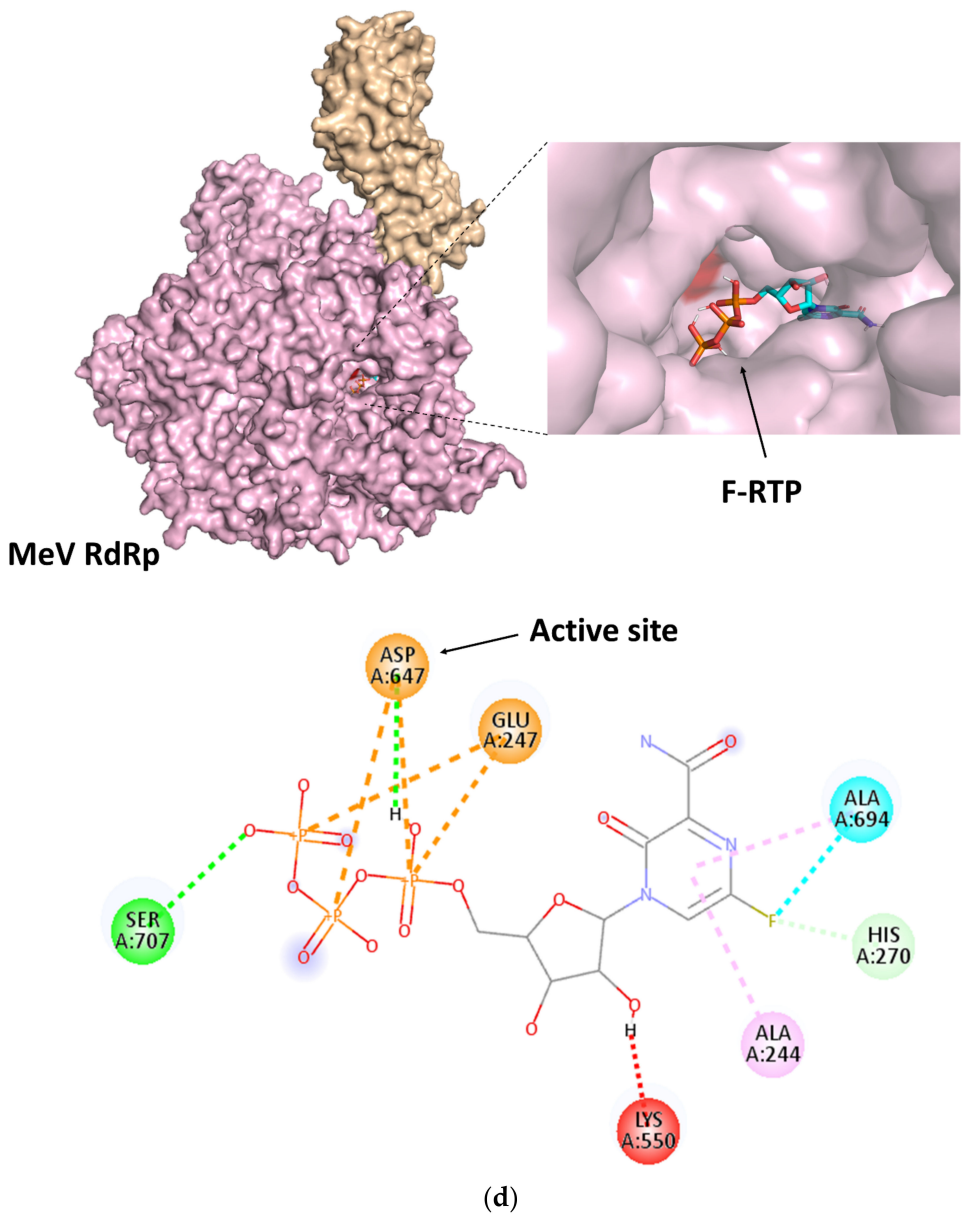

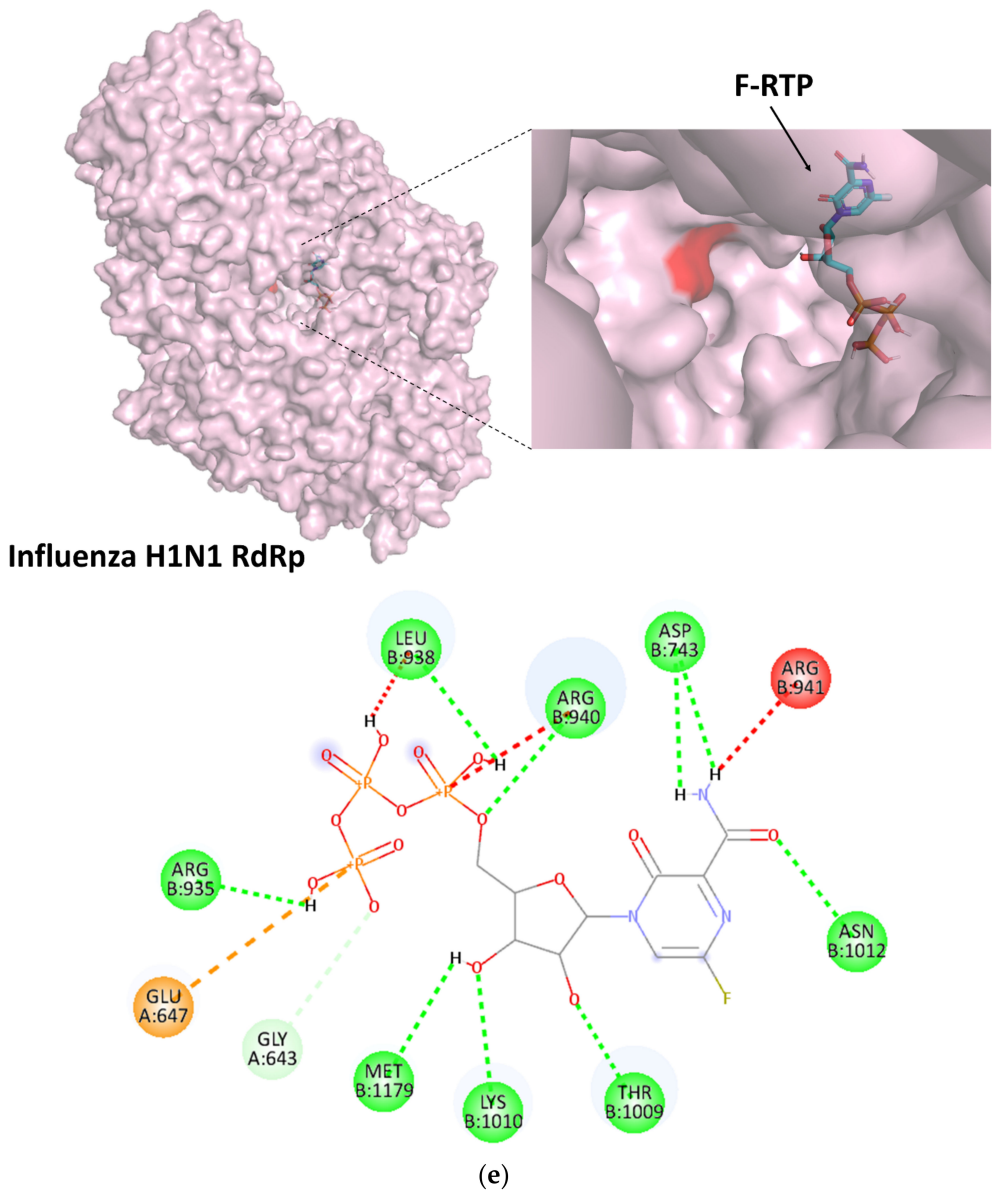

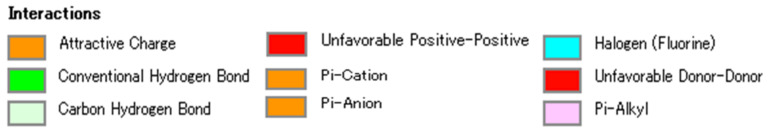
Diagram illustrating the binding conformations and interactions of favipiravir ribofuranosyl-5′-triphosphate (F-RTP) with (**a**) human metapneumovirus (HMPV) RNA-dependent RNA polymerase (RdRp), (**b**) respiratory syncytial virus (RSV) RdRp, (**c**) mumps virus (MuV) RdRp, (**d**) measles virus (MeV) RdRp, and (**e**) influenza H1N1 RdRp. The red color on the surface of various proteins indicates active sites.

First, we performed molecular docking studies using F-RTP and various proteins alone and analyzed how they create interactions. As shown in Figure 2a–c, the triphosphate group of F-RTP formed electrostatic interactions (attractive charge) and conventional hydrogen bonds with the active sites in the RdRp proteins of HMPV, RSV, and MuV (Asp745 and Asn746, Asp811 and Asn812, and Asp665 and Asn666, respectively).

Similarly, the docking simulation of MeV RdRp showed that the Asp647 residues at the active site also interacted with F-RTP with electrostatic interactions (attractive charge) and conventional hydrogen bonds, but the asparagine residue of the active (Asn648) sites was not involved in the interactions (Figure 2d). An unfavorable interaction (donor–donor) was formed with Lys550 in the MeV RdRp protein. The attractive forces involved in sites other than the active sites were carbon-hydrogen bonds, halogen interactions, electrostatic interactions (attractive charge, pi-cation, and pi-anion), and hydrophobic interactions (pi-alkyl) in the four RdRp proteins (HMPV, RSV, MuV, and MeV).

The interacting residues other than the active sites were alanine, arginine, aspartic acid, glutamine, glutamic acid, glycine, histidine, leucine, lysine, serine, and threonine. The intermolecular distances between F-RTP and amino acid residues in the RdRp active sites were 2.1–5.5 Å (Table 1). The calculated binding energies between F-RTP and the RdRp proteins were as follows: HMPV, −7.8 kcal/mol; RSV, −6.3 kcal/mol; MuV, −6.4 kcal/mol; and MeV, −6.7 kcal/mol (Table 1).

F-RTP did not bind to the active site; rather, it bound to the RNA synthesis pathway of the influenza H1N1 RdRp protein (Figure 2e). Molecular binding was mediated through hydrogen bonds (carbon and conventional) with Gly643, Asp743, Arg935, Leu938, Arg940, Thr1009, Lys1010, Asn1012, and Met1179 and electrostatic interactions (attractive charge) with Glu647. Unfavorable interactions (donor–donor and positive–positive) were also formed with Leu938, Arg940, and Arg941. The estimated binding energy between F-RTP and the influenza H1N1 RdRp protein was −7.4 kcal/mol (Table 1).

### 3.3. Molecular Interactions among Various RdRp Proteins, NTP, Magnesium Ions, and F-RTP

We also conducted molecular docking studies among various RdRp proteins, NTP, magnesium ions, and F-RTP to clarify the conformations and interactions of F-RTP in the RNA synthesis process by RdRp. 

**Figure 3:**
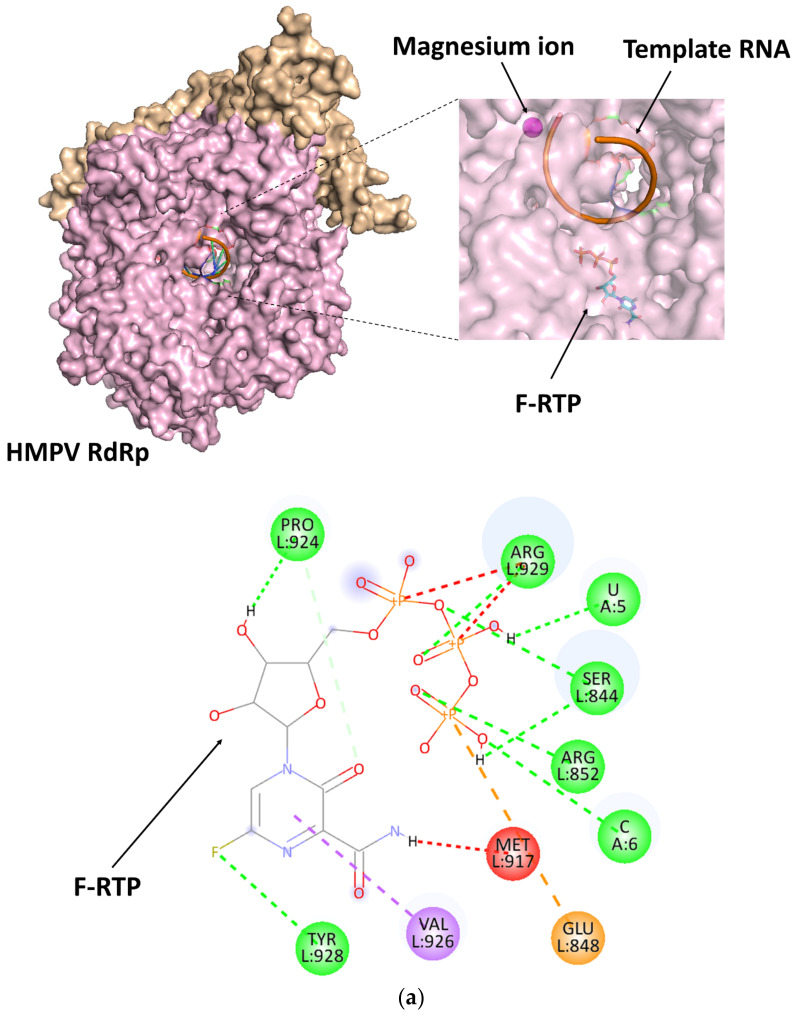

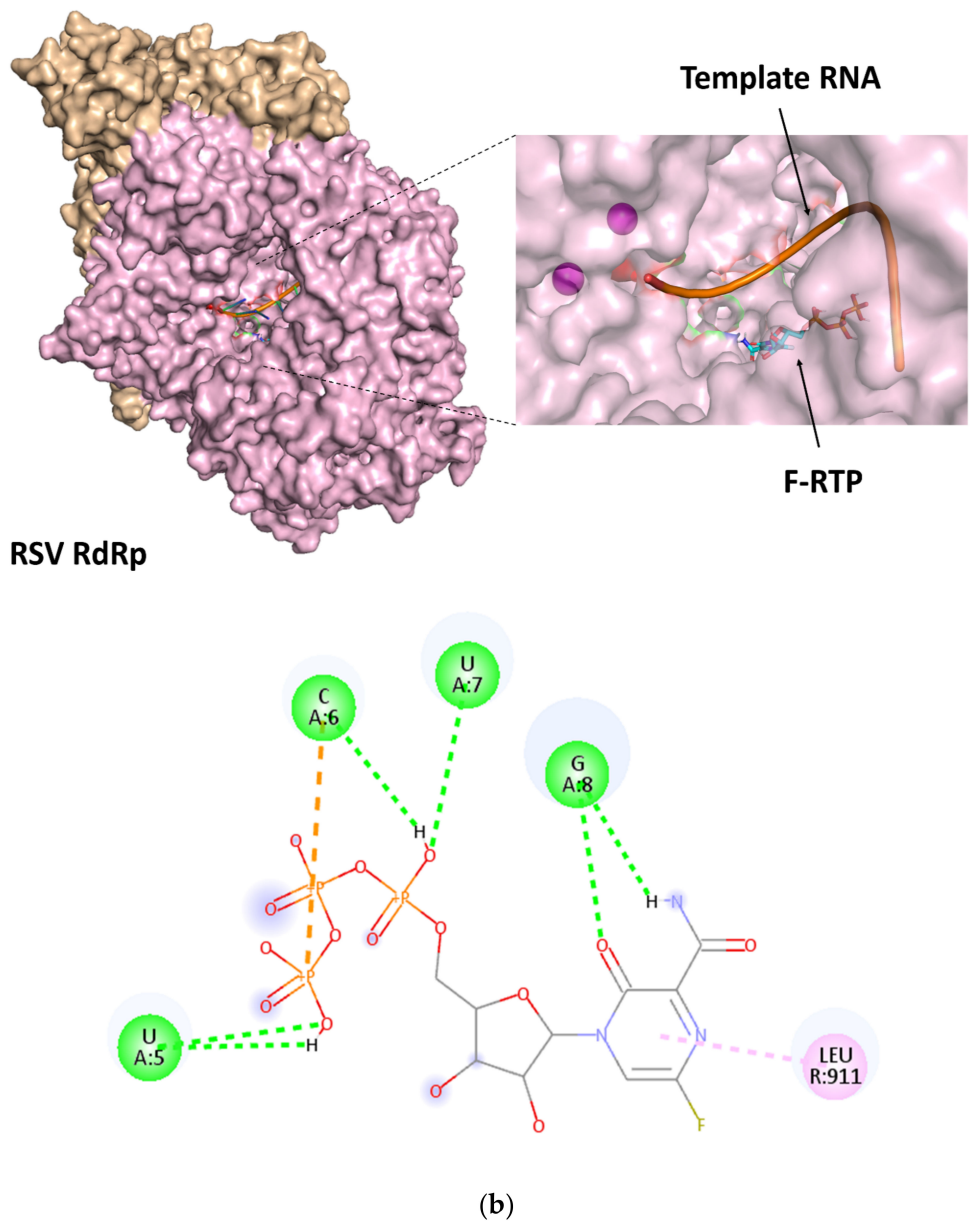

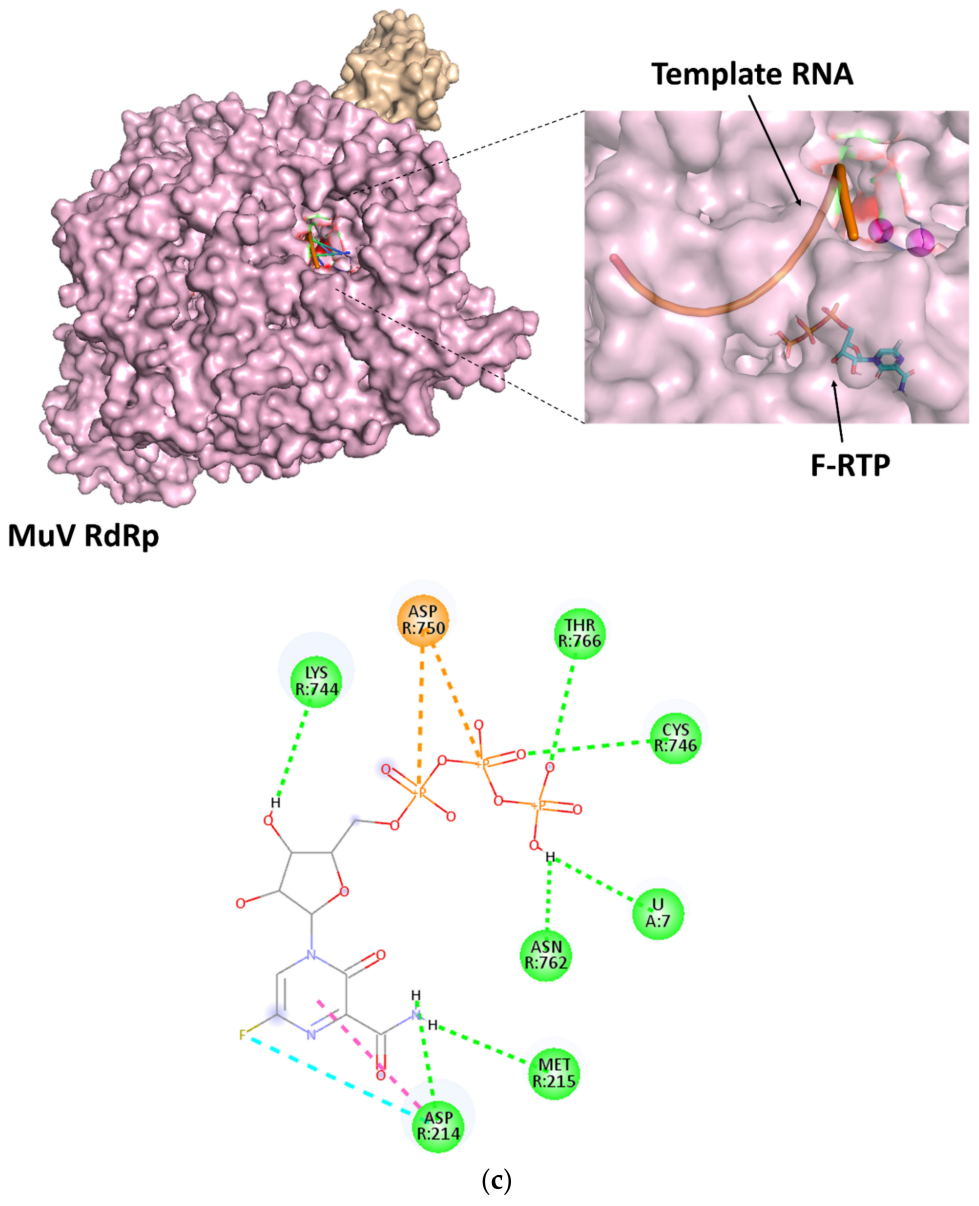

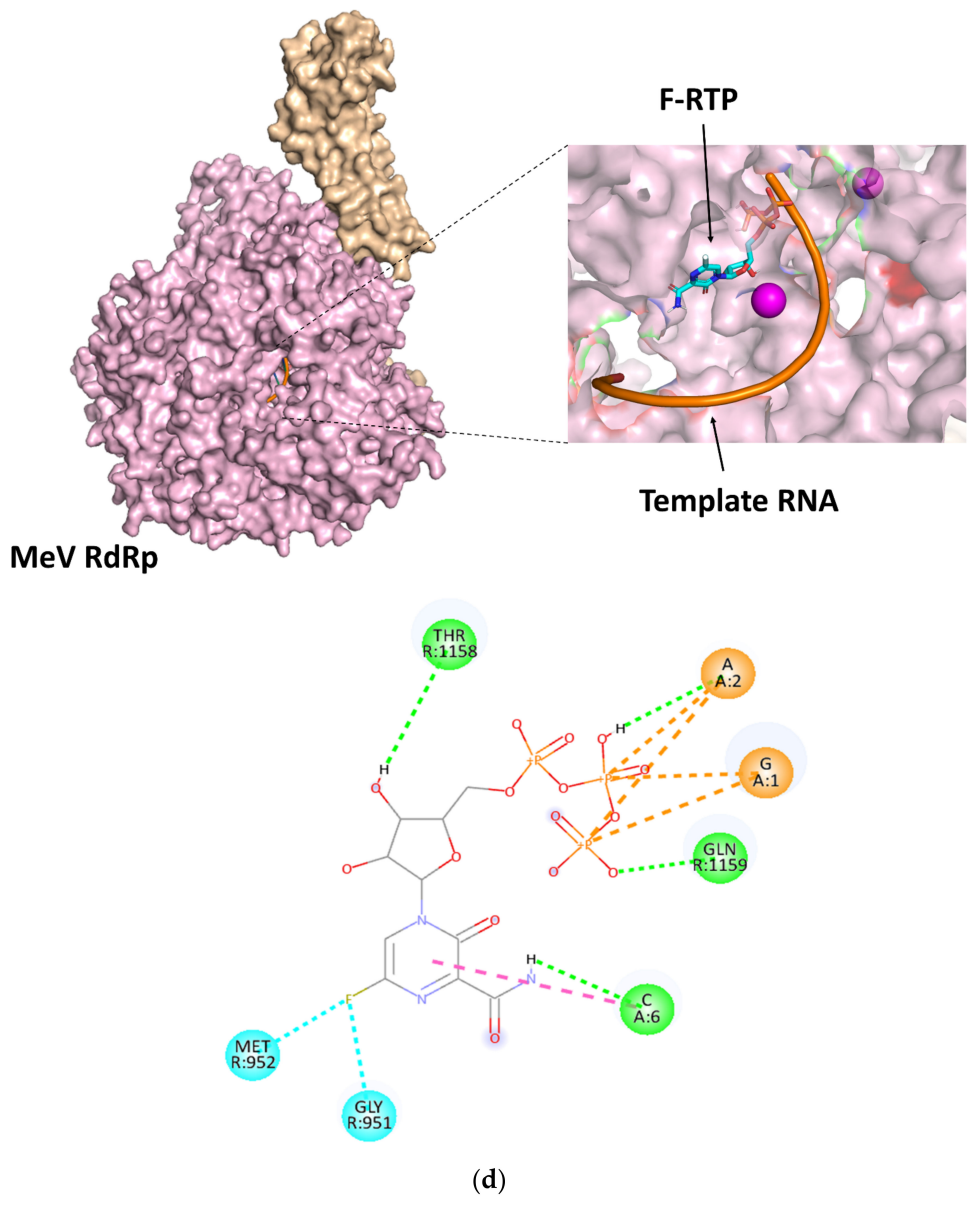

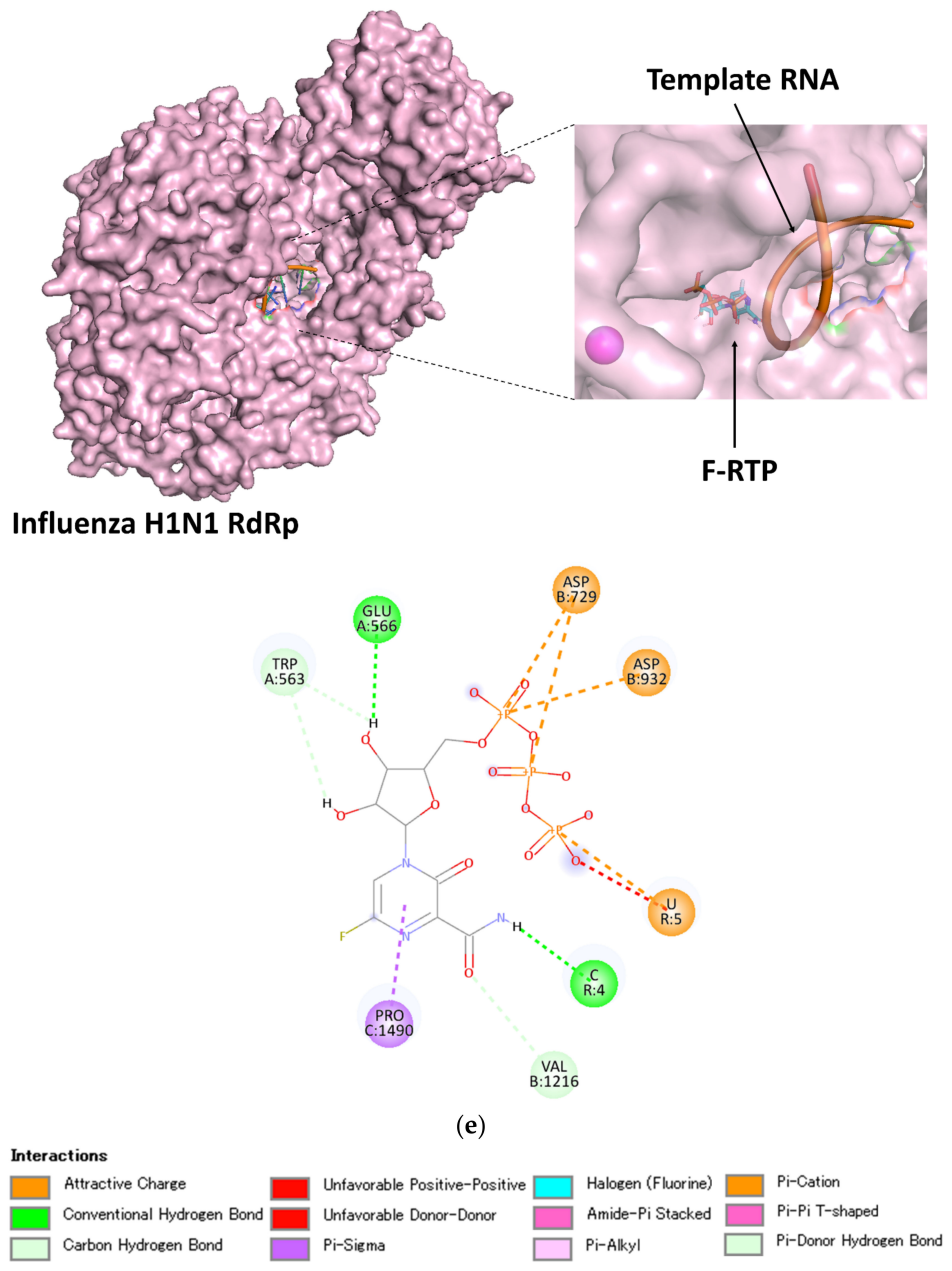
Diagram illustrating the binding conformations and interactions among favipiravir ribofuranosyl-5′-triphosphate (F-RTP), nucleotide triphosphate (NTP), magnesium ions, and (**a**) human metapneumovirus (HMPV) RNA-dependent RNA polymerase (RdRp), (**b**) respiratory syncytial virus (RSV) RdRp, (**c**) mumps virus (MuV) RdRp, (**d**) measles virus (MeV) RdRp, and (**e**) influenza H1N1 RdRp. The active sites on proteins are shown in red. Magnesium ions that bound to the RdRp are colored magenta.

As shown in Figure 3, in all RdRp proteins, F-RTP bound to the template RNA; however, the interaction forces varied: HMPV: electrostatic interactions (attractive charge), hydrogen bonds (carbon and conventional), pi-sigma interactions, and unfavorable interactions (donor–donor and positive–positive); RSV: electrostatic interactions (pi-cation), hydrogen bonds (carbon and conventional), and hydrophobic interactions (pi-alkyl); MuV: electrostatic interactions (attractive charge), halogen bonds, hydrogen bond (conventional), and hydrophobic interactions (amide-pi stacked); MeV: electrostatic interactions (attractive charge and pi-cation), halogen bonds, hydrogen bonds (conventional), and hydrophobic interactions (pi–pi T-shaped); influenza H1N1, electrostatic interactions (attractive charge), hydrogen bonds (carbon, conventional, and pi-donor), pi-sigma interaction, and unfavorable interaction (acceptor–acceptor).

The interacting sites of the template RNA also varied and included all four types of bases. Magnesium ions did not interact with F-RTP in the docking simulation. The binding energies between F-RTP and the RdRp proteins of HMPV, RSV, MuV, MeV, and influenza H1N1 were calculated as −7.1, −7.7, −7.0, −7.7, and −7.6 kcal/mol, respectively.

### 3.4. Rescoring the Structure of Protein-Ligand Complex

We rescored the docking poses using PoseScore of LigScore to validate the present docking simulations. Due to the validation, some models, including HMPV with NTP, MuV with NTP, and influenza H1N1 with NTP, changed (Supplemental Figure S1). As a result, the optimal models were determined (Figure 2 and Figure 3). In the absence of NTP, the docking poses of favipiravir binding to RdRp of HMPV, RSV, MuV, MeV, and influenza H1N1 were 1st, 4th, 11th, 4th, and 11th, respectively, and in the presence of NTP and magnesium ions, they were 2nd, 7th, 3rd, 15th, and 1st, respectively, based on the LigScore. The best-scored model of LigScore except for HMPV and influenza H1N1 with NTP did not bind to the RdRp active sites or the template RNA (Supplementary Figure S2).

## 4. Discussion

In the present study, we performed a detailed analysis of the molecular interactions between various RdRps (HMPV, RSV, MuV, MeV, and influenza virus subtype A (H1N1)) and F-RTP (the favipiravir active form). As a result, we found that F-RTP bound to the active sites (HMPV, RSV, MuV, and MeV) or adjacent to the RdRp active sites (influenza virus). Moreover, F-RTP was incorporated into the replicating RNA molecules of all viruses under the presence of NTP and magnesium ions. These results suggested that F-RTP may act as not only an inhibitor of active sites in the RdRp complex but also an inhibitor of the replication of RNA. To the best of our knowledge, this may be the first observation of these interactions in HMPV, RSV, MuV, and MeV based on an in silico study.

HMPV, RSV, MuV, and MeV are associated with major common diseases. Thus, the need for antiviral drugs that treat these infectious diseases is growing; however, at the time of writing this report, no effective antivirals exist [4,6,7,8]. Recent studies suggested the possible drug repositioning of molnupiravir (an anti-influenza virus drug) for the treatment of coronavirus disease 2019 (COVID-19) [44]. Moreover, previous reports suggested that favipiravir was also an effective antiviral agent for various viruses, including HMPV, RSV, MuV, and MeV, based on an in vitro study [14,15]. However, the precise molecular interactions between favipiravir and their RdRp are not known. Thus, we performed a detailed analysis of these molecular interactions in silico.

The present data showed that the F-RTP could bind to the RdRp active sites of HMPV, RSV, MuV, and MeV (Figure 2). However, F-RTP did not directly bind to the RdRp active sites in influenza H1N1; rather, it bound to the RNA synthesis pathway. Most antiviral nucleic acid analogs act as the competitive inhibition of RdRp due to incorporation into the replicating viral genome [45]. Favipiravir also inhibits the RdRp competitively [9]. In contrast, previous reports showed that favipiravir directly affects the activity of RdRp partially [14,46,47]. However, the mechanisms of direct inhibition of RdRp are not exactly known. In the present study, we found that F-RTP binding to the RdRp active site may lead to a decrease of RdRp activity.

Our previous report showed that F-RTP binds near the tunnel of influenza RdRp and could bind to RdRp active sites in coronavirus [48]. This result is consistent with the present study, whereas we newly showed that the RdRp active sites residues in coronavirus (serine and two aspartic acids) were different from those in HMPV, RSV, MuV, and MeV. Therefore, we analyzed the amino acid residues and interactions involved in the binding between them in more detail. As shown in Figure 2, the aspartic acid residue in the RdRp active sites interacted with the triphosphate group of F-RTP in HMPV, RSV, MuV, and MeV.

These interactions may be responsible for electrostatic interactions and hydrogen bonds. It is suggested that aspartic acid residue is a common amino acid in various RNA viral RdRp active sites because this amino acid interacts with magnesium ion as an essential component for viral genome replication [49,50]. Taken together, F-RTP may be associated with the inhibition of viral genome replication when aspartic acid residue is present in RdRp active sites. To the best of our knowledge, this is the first study to show interactions between F-RTP and aspartic acid residue in the RdRp active site.

Favipiravir was developed as an antiviral agent for the treatment of influenza. The agent has been reported to be metabolized by hypoxanthine-guanine phosphoribosyltransferase, resulting in the active form (F-RTP) [10]. F-RTP can be incorporated into the replicating viral genome [51]. Due to these procedures, the termination or mismatch occurs in the replicating viral genome [9]. Such mechanisms can contribute to antiviral effects.

Our in silico data suggested that F-RTP was incorporated into various replicating viral genomes (e.g., HMPV, RSV, MuV, MeV, and influenza virus) (Figure 3). Our data, as well as those from previous studies, also suggested that the F-RTP may inhibit these viral replications [10,11,14,15]. However, the present data could not show whether F-RTP elicited termination or mismatch in the replicating viral genomes. Further, the interacting sites of template RNA varied in this study.

However, the cryo-EM structure and in vitro studies show that F-RTP is mis-incorporated into the replicating viral genomes as purine nucleotides [10,52,53]. In the present docking simulation, the small number of template RNA bases and the absence of competitive NTP substrates may affect our results. To better understand the distinct results between previous studies and our study, further research may be needed.

Previous in vitro studies showed distinct data regarding the EC50 or EC90 of favipiravir [14,15,54]. These different data may be partially responsible for the distinct principles of the examination. Thus, we could not simply compare the effective doses of the agent in these in vitro studies with the binding energy of the present study. In addition, the present study could not estimate the F-RTP dose required for the inhibition of viral genome replication because we could not modulate the doses of various components (e.g., F-RTP, magnesium ion, and NTP). This may be one limitation of the present study.

The best-scored docking pose does not always reflect the actual binding pose. Thus, further analysis may be needed to be performed to validate the docking protocol, one of which is cross-docking [55]. However, crystallographic RdRp structures that are bound to other ligands and cocrystallized favipiravir were not available. An alternative approach to improve the accuracy of docking simulations is to rescore the top-ranked docking molecules using another scoring function [56,57].

Previous reports suggested that LigScore uses atomistic distance-dependent statistical scoring functions, which is different from the scoring function of AutoDock Vina [40,58]. Hence, we used LigScore as a rescoring method to validate the docking protocol in the present study. The rescoring analysis showed the accuracy of the docking simulation, with high rankings for many viruses. However, the rankings of certain viruses (influenza H1N1, MuV, RSV with NTP, and MeV with NTP) were low. Further improvement of the accuracy of the docking simulation will be a subject for future study. This is a limitation of the present study.

Finally, our simulation could not reveal the process of F-RTP incorporated into the growing viral genomes and causing a termination or lethal mutagenesis in them in the presence of NTP substrates. Previous in vitro studies showed that a conserved lysine residue in various virus RdRp may be responsible for the antiviral effects of favipiravir [11,59]. However, we could not show this in the present study because this lysine residue is not located on the RdRp active sites and has a key role in the process of F-RTP binding to them [11,59]. At present, advanced calculation technologies and molecular dynamics simulations allow us to show the processes of various small molecules recognition by the target proteins as a dynamic image [60,61].

However, extremely high-performance computer systems with suitable software are required for these analyses, and only limited laboratories can perform these. Further studies may be needed in the future.

## 5. Conclusions

We performed a precise analysis of the molecular interaction between favipiravir and the RdRp of HMPV, RSV, MuV, MeV, and influenza virus using in silico methods. Our study showed that F-RTP bound to the RdRp active sites in HMPV, RSV, MuV, and MeV. Moreover, the triphosphate group of F-RTP interacted with the aspartic acid residue of the RdRp active sites in all of these viruses.

However, F-RTP did not directly bind to the RdRp active sites in influenza virus but instead bound adjacent to it. Furthermore, we showed that F-RTP was incorporated into the nascent viral RNA genome in the presence of NTP and magnesium ions. The results indicated that favipiravir exhibits two different molecular interactions in various viruses: RdRp active site inhibition and/or genome replication inhibition.

## Figures and Tables

**Table 1:** Interactions of F-RTP with RdRp active sites and binding energy in various viruses.

Virus	Residue	Force	Distance (Å)	Binding Energy (kcal/mol)
HMPV	Asp745	Electrostatic interaction	3.67, 5.52	−7.8
	Asn746	hydrogen bond	2.42	
RSV	Asp811	Hydrogen bond, electrostatic interaction	2.26, 4.73	−6.3
	Asn812	hydrogen bond	2.60	
MuV	Asp665	Hydrogen bond, electrostatic interaction	2.36, 4.63	−6.4
	Asn666	hydrogen bond	2.05	
MeV	Asp647	Hydrogen bond, electrostatic interaction	2.37, 3.76, 5.01	−6.7
Influenza H1N1	None	None	None	−7.4

## Data Availability

The data presented in this study are available in the article.

## Supplementary Materials

The following are available online at https://www.mdpi.com/article/10.3390/v14020338/s1, Figure S1: Comparison between models selected by autodock alone and those changed by the addition of LigScore. Figure S2: The best-scored model of LigScore.
