# Supplementary material for: Detailed Analyses of Molecular Interactions between Favipiravir and RNA Viruses In Silico
Source: Viruses. 2022 Feb 7;14(2):338. doi: 10.3390/v14020338 (PMC8879546; doi:10.3390/v14020338)
Supplement: Supplementary file 1 [file viruses-14-00338-s001.zip › viruses-1547807-supplementary.pdf]

Supplementary Figure

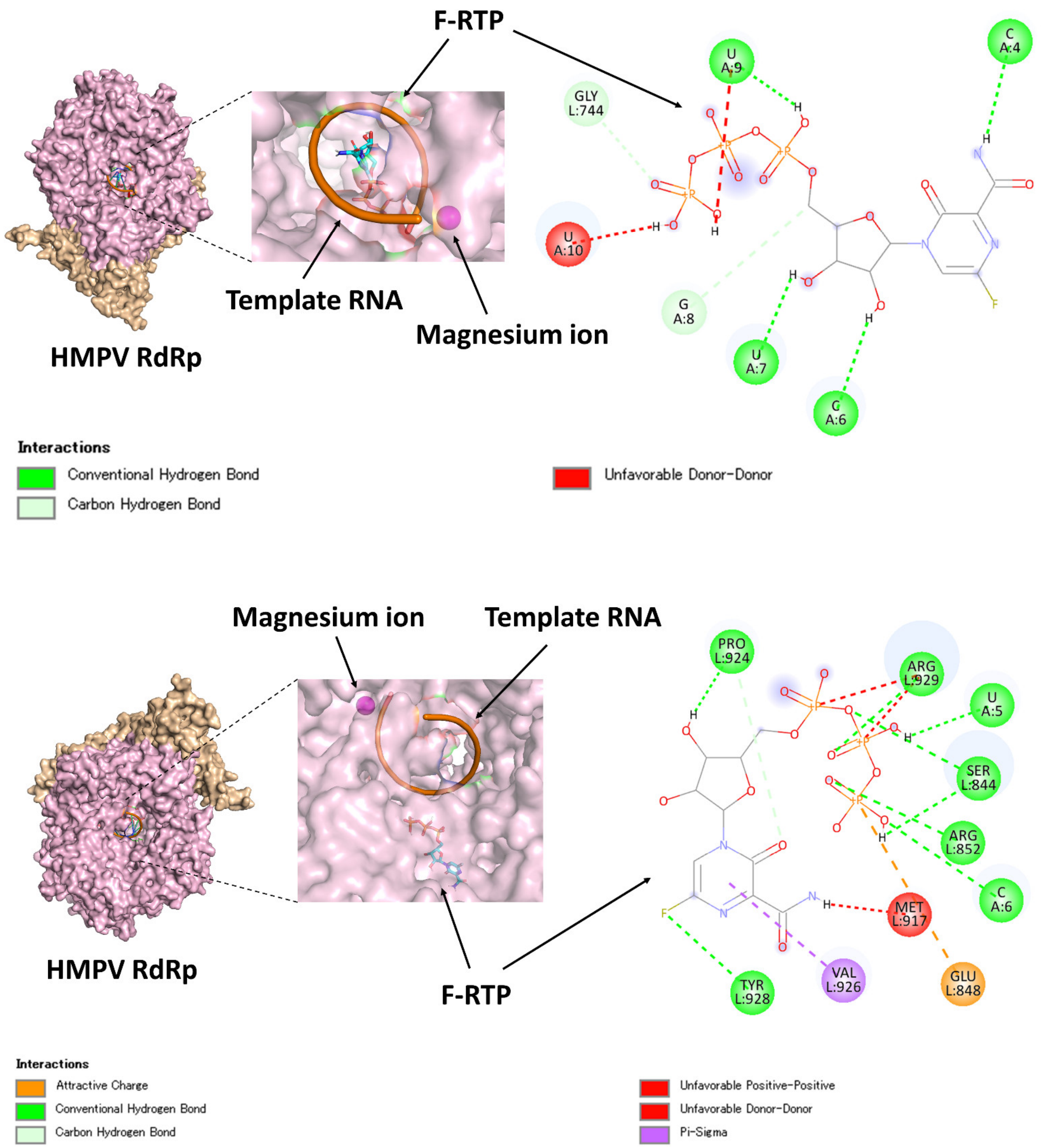

(a)

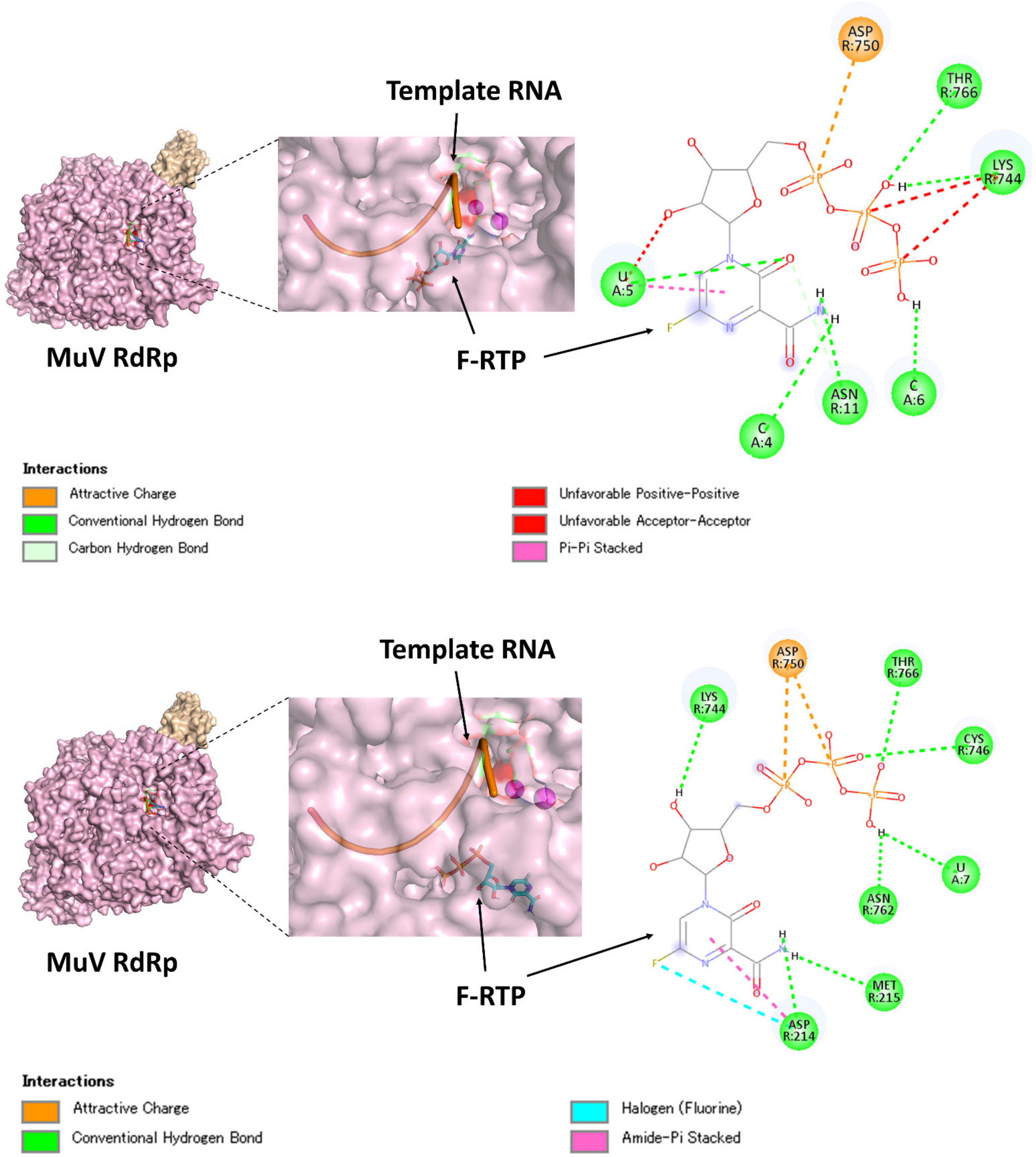
Template RNA
MuV RdRp
F-RTP
ASP R:750
THR R:766
LYS R:744
U A:5
ASN R:11
C A:6
C A:4
Interactions
Attractive Charge
Conventional Hydrogen Bond
Carbon Hydrogen Bond
Unfavorable Positive-Positive
Unfavorable Acceptor-Acceptor
Pi-Pi Stacked
Template RNA
MuV RdRp
F-RTP
ASP R:750
THR R:766
LYS R:744
CYS R:746
U A:7
ASN R:762
MET R:215
ASP R:214
Interactions
Attractive Charge
Conventional Hydrogen Bond
Halogen (Fluorine)
Amide-Pi Stacked

(b)

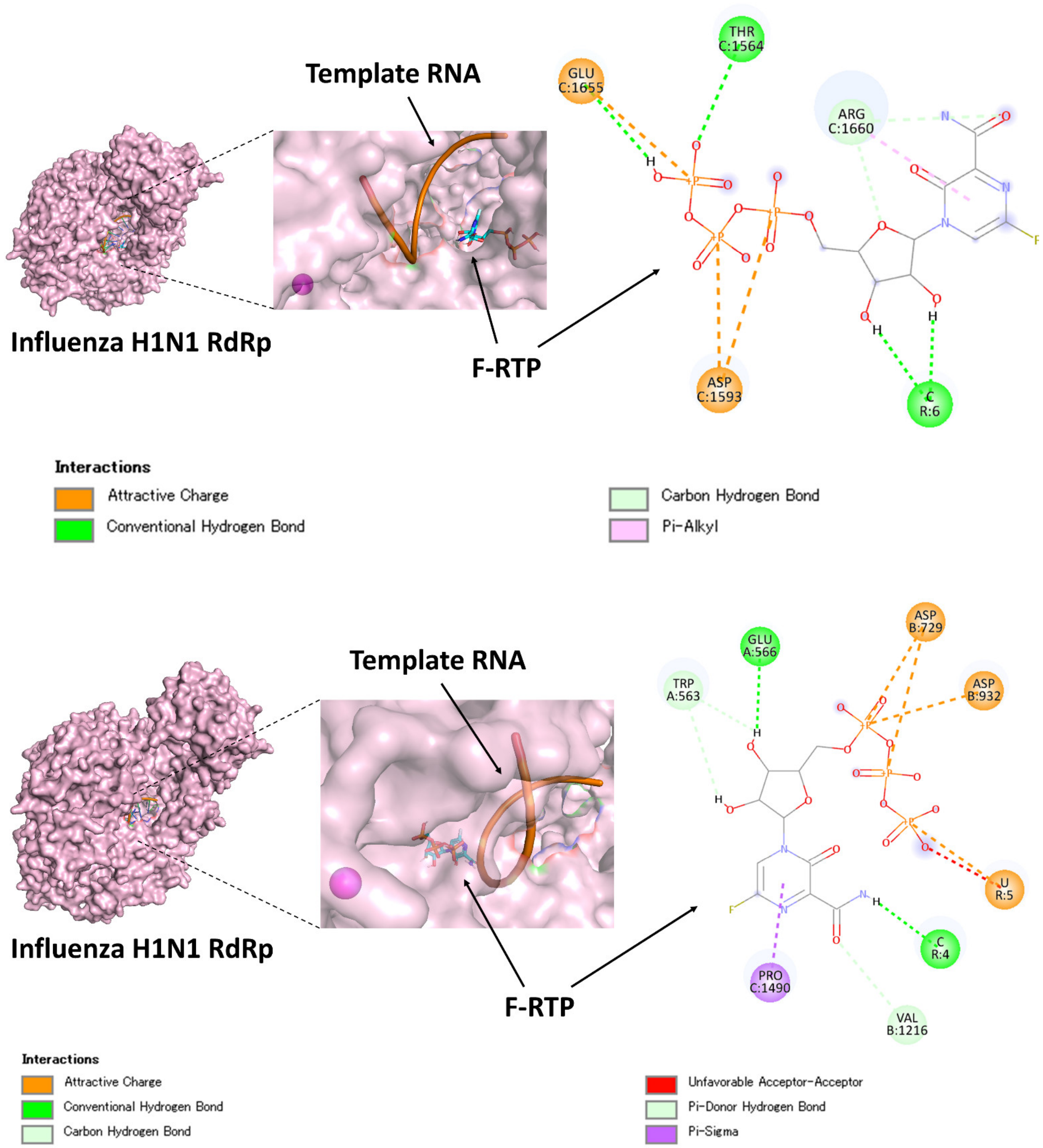

(c)

Supplementary Figure S1. Comparison between models selected by autodock alone (the upper figure) and those changed by the addition of LigScore (the lower figure). Each Diagram illustrates the binding conformations and interactions among favipiravir ribofuranosyl-5′-triphosphate (F-RTP), nucleotide triphosphate (NTP), magnesium ions, and (**a**) human metapneumovirus (HMPV) RNA-dependent RNA polymerase (RdRp), (**b**) mumps virus (MuV) RdRp, and (**c**) influenza H1N1 RdRp. The active sites on proteins are shown in red. Magnesium ions that bound to the RdRp are colored magenta.

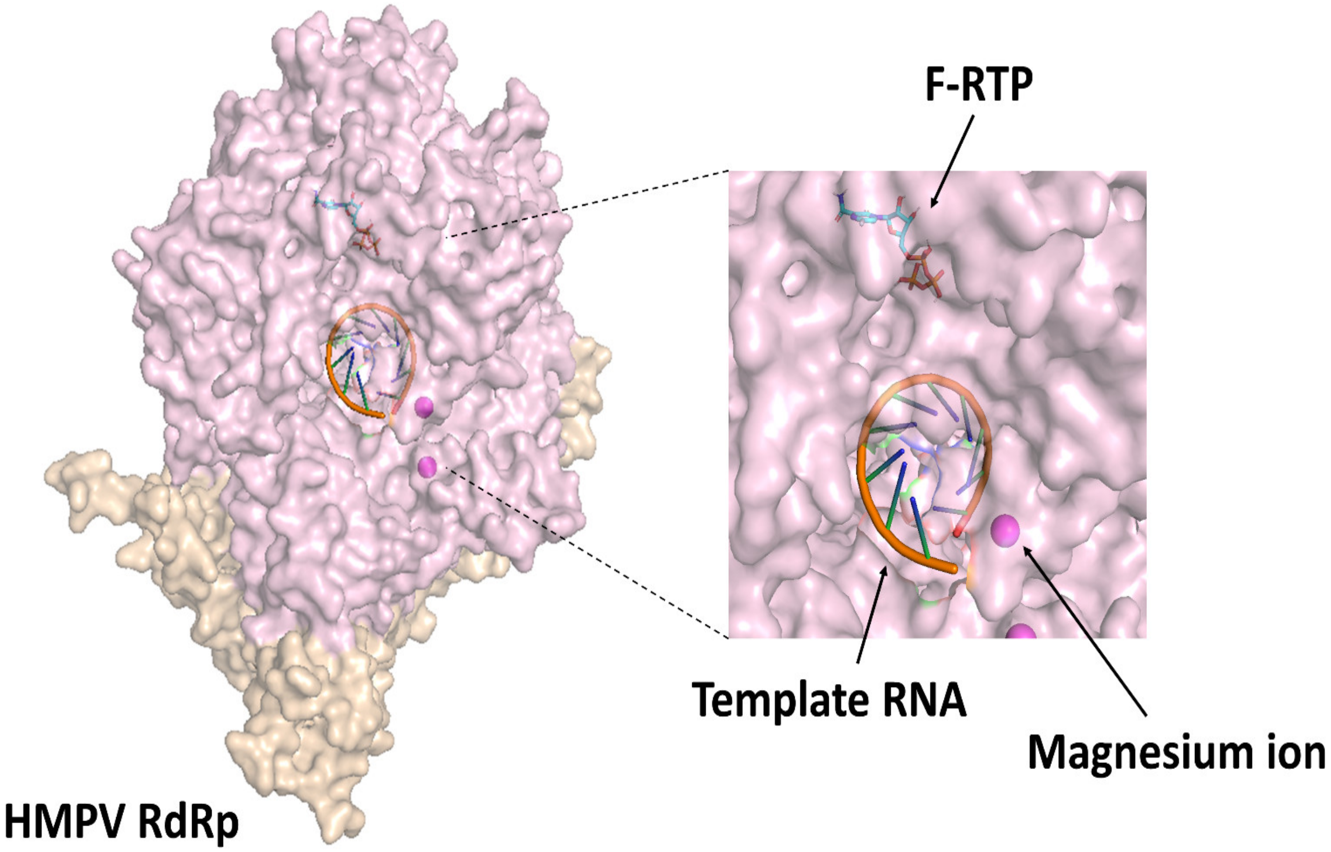

(a)

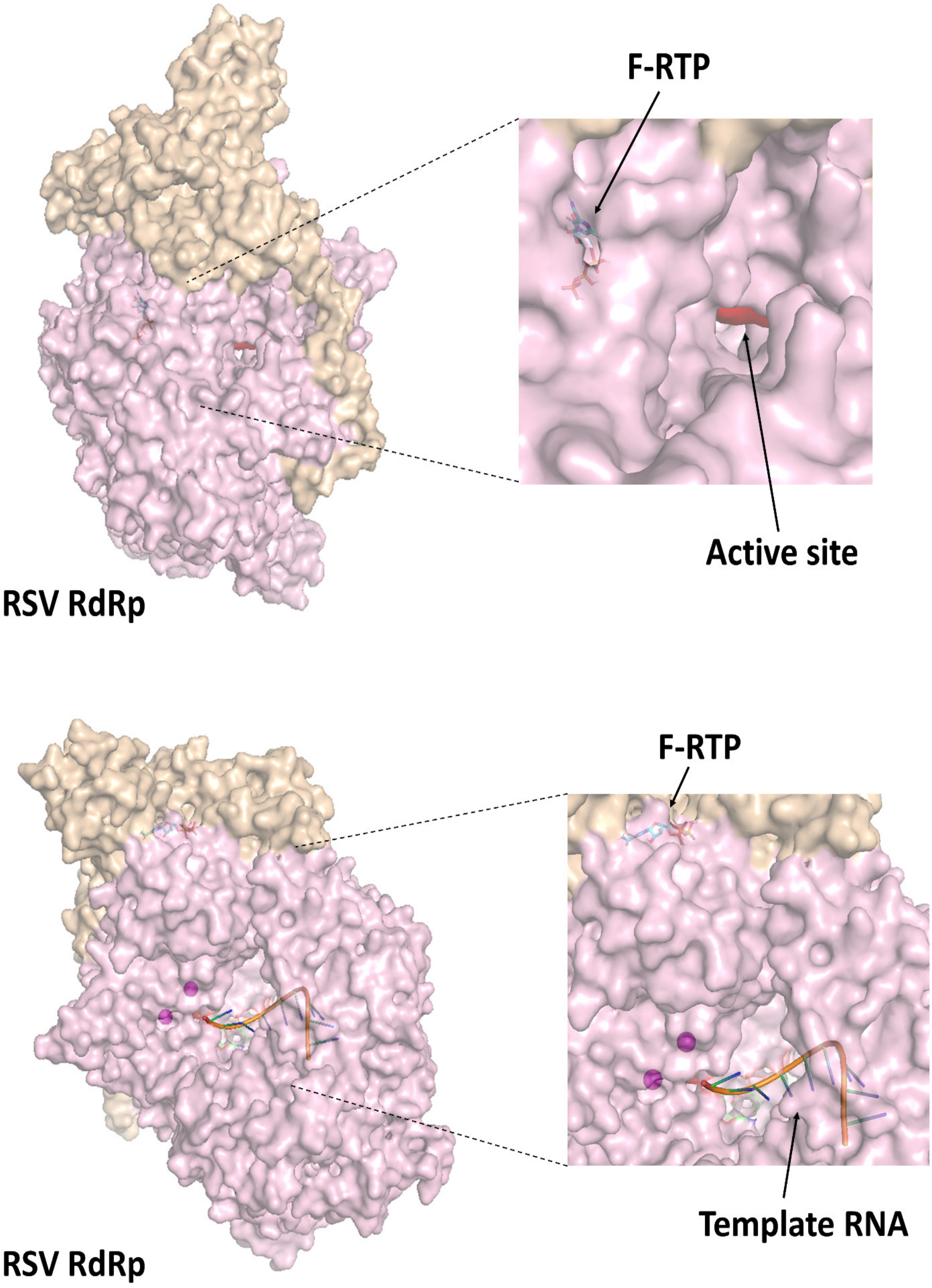

(b)

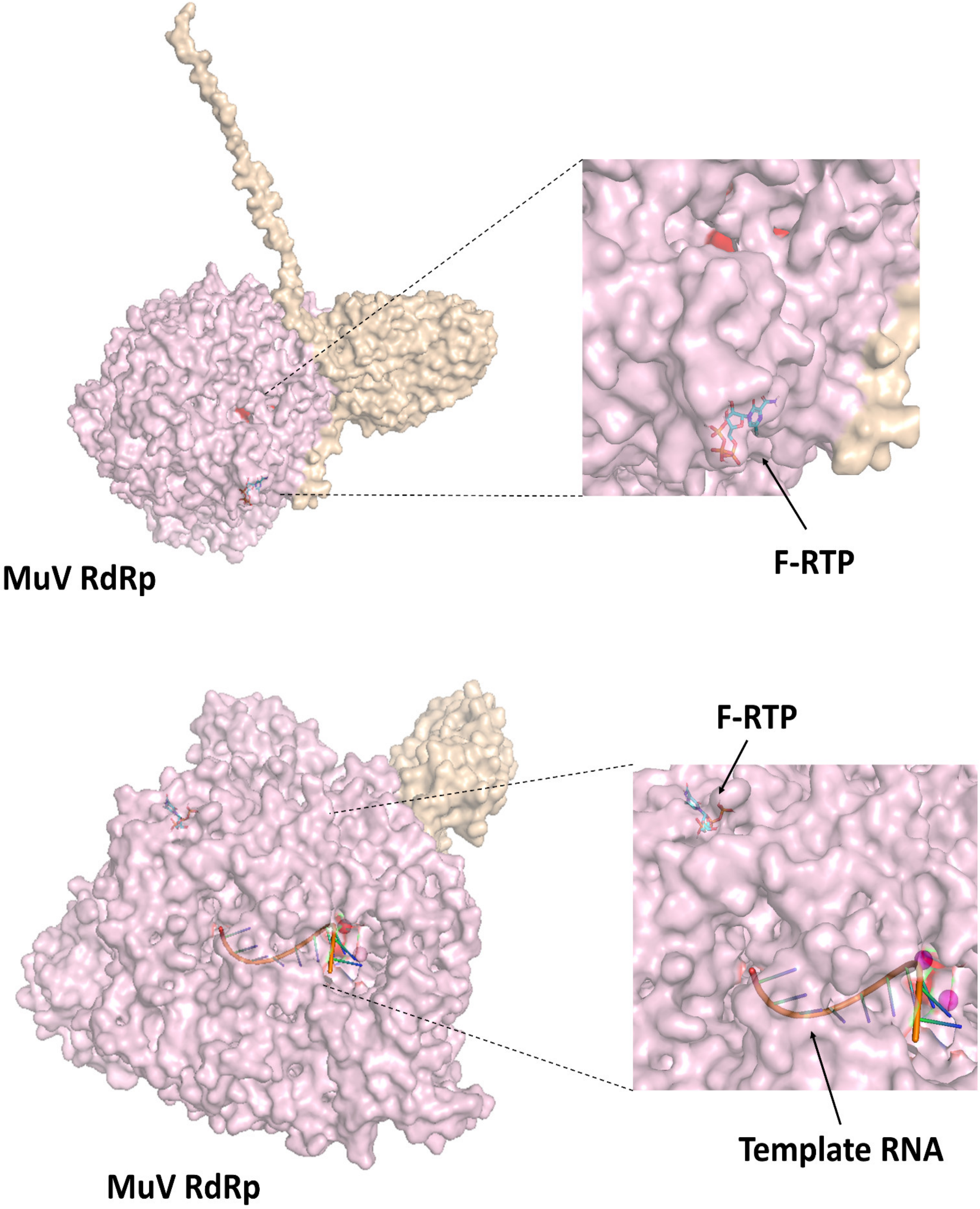

(c)

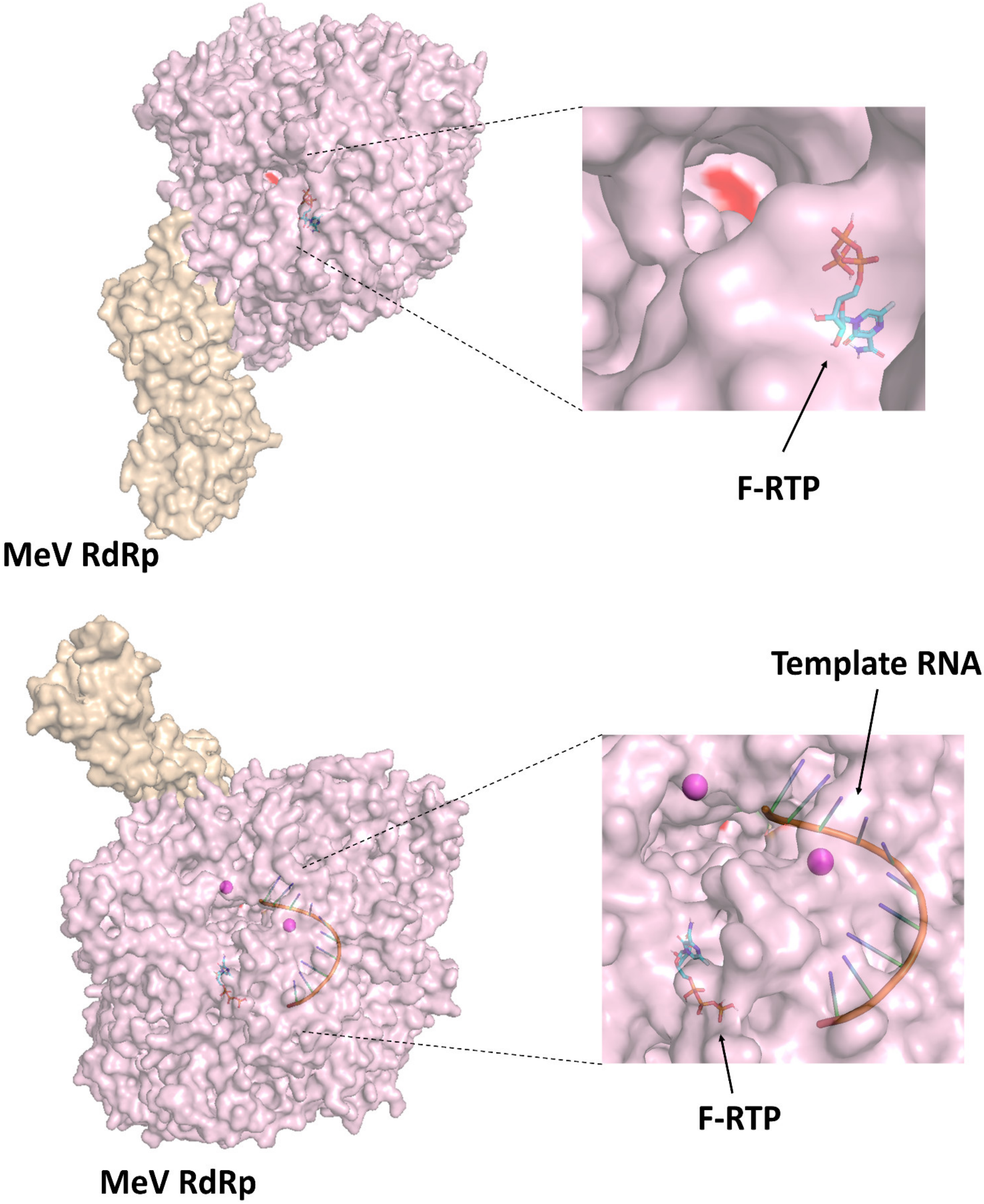

(d)

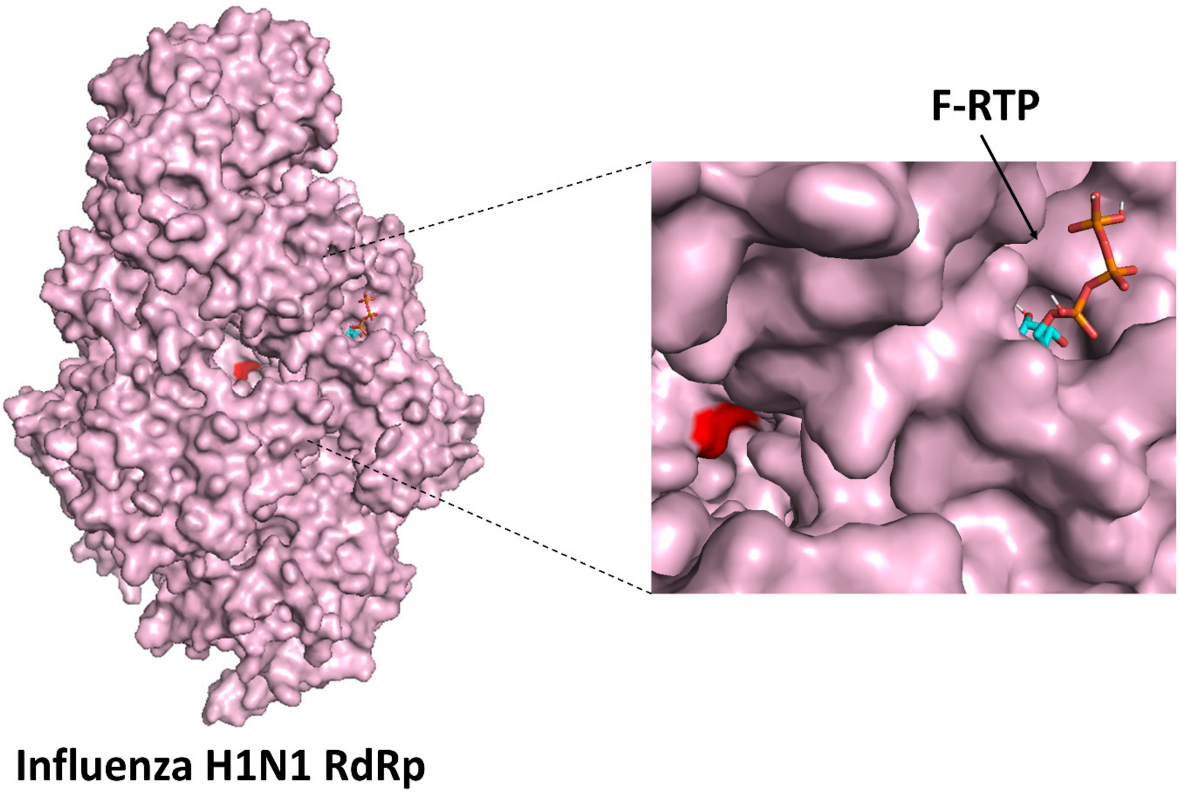

(e)

Supplementary Figure S2. Diagram illustrating the best-scored binding conformations of favipiravir ribofuranosyl-5′-triphosphate (F-RTP) with (**a**) human metapneumovirus (HMPV) RNA-dependent RNA polymerase (RdRp), (**b**) respiratory syncytial virus (RSV) RdRp, (**c**) mumps virus (MuV) RdRp, (**d**) measles virus (MeV) RdRp, and (**e**) influenza H1N1 RdRp based on LigScore. The red color on the surface of various proteins indicates active sites. Magnesium ions that bound to the RdRp are colored magenta.
